# A social ecological model of willingness to participate in HIV vaccine trials among men who have sex with men in Chennai and Mumbai, India

**DOI:** 10.1186/1742-4690-9-S2-P111

**Published:** 2012-09-13

**Authors:** V Chakrapani, PA Newman, J Jerajani, M Shanmugam, N Singhal

**Affiliations:** 1 Centre for Sexuality and Health Research and Policy; Humsafar Trust, Chennai and Mumbai, India; 2 University of Toronto, Toronto, Canada; 3 The Humsafar Trust, Mumbai, India

## Background

Recruitment of low- and middle-income country volunteers from most-at-risk-populations in HIV vaccine trials is essential to vaccine development. In India, men who have sex with men (MSM), at 20-fold higher risk for HIV infection than the general population, are a crucial population for recruitment. Research on willingness to participate (WTP) in HIV vaccine trials has focused predominantly on individual-level determinants (e.g., safety concerns, mistrust, altruism) in high-income countries. We used a social ecological framework to explore multi-level determinants of WTP among MSM in India.

## Methods

We conducted 9 focus groups (n=49) with low socioeconomic MSM (aged 20-46 years, mean=28), including peer outreach workers, in Chennai (in Tamil) and Mumbai (in Marathi/Hindi), and 7 key informant interviews with MSM community leaders and healthcare professionals. Focus groups/interviews were recorded, transcribed and translated into English. Two independent bilingual investigators conducted narrative thematic analysis using line-by-line coding and a constant comparative method, with member-checking by community representatives.

## Results

Structural-level determinants of WTP included poverty, compensation for trial-related harms and having financially-dependent family members. Community-level factors were negative societal attitudes about same-sex sexuality and community engagement in HIV vaccine research. Interpersonal factors included anticipated peer and familial reactions and perceived HIV stigma. Individual-level determinants of WTP included knowledge about vaccines, openness about one’s sexuality and altruistic beliefs.

## Conclusion

WTP in HIV vaccine trials among MSM in India is associated with multi-level factors. Ubiquitous interpersonal and community-level concerns characteristic of the sociocultural context may complicate individual-focused conceptualizations of WTP. Interventions to reduce stigma and discrimination against MSM and PLHIV, capacity-building of MSM service organizations, and sustained and transparent communications tailored to the knowledge, concerns and educational level of local communities may support meaningful engagement of MSM in HIV vaccine trial preparedness. Attention to providing fair yet non-coercive compensation and healthcare benefits are warranted to support ethical conduct of trials.

